# Active triggering control of pneumatic rehabilitation gloves based on surface electromyography sensors

**DOI:** 10.7717/peerj-cs.448

**Published:** 2021-04-19

**Authors:** Yongfei Feng, Mingwei Zhong, Xusheng Wang, Hao Lu, Hongbo Wang, Pengcheng Liu, Luige Vladareanu

**Affiliations:** 1 Faculty of Mechanical Engineering & Mechanics, Ningbo University, Ningbo, Zhejiang Province, China; 2 Robotics and Mechatronics Department, Institute of Solid Mechanics of the Romanian Academy, Bucharest, Bucharest, Romania; 3 Academy for Engineering & Technology, Fudan University, Shanghai, Shanghai, China; 4 Department of Computer Science, University of York, York, York, United Kingdom

**Keywords:** Hand rehabilitation, Pneumatic rehabilitation gloves, Surface electromyography, Active trigger control system, Back propagation neural network

## Abstract

The portable and inexpensive hand rehabilitation robot has become a practical rehabilitation device for patients with hand dysfunction. A pneumatic rehabilitation glove with an active trigger control system is proposed, which is based on surface electromyography (sEMG) signals. It can trigger the hand movement based on the patient’s hand movement trend, which may improve the enthusiasm and efficiency of patient training. Firstly, analysis of sEMG sensor installation position on human’s arm and signal acquisition process were carried out. Then, according to the statistical law, three optimal eigenvalues of sEMG signals were selected as the follow-up neural network classification input. Using the back propagation (BP) neural network, the classifier of hand movement is established. Moreover, the mapping relationship between hand sEMG signals and hand actions is built by training and testing. Different patients choose the same optimal eigenvalues, and the calculation formula of eigenvalues’ amplitude is unique. Due to the differences among individuals, the weights and thresholds of each node in the BP neural network model corresponding to different patients are not the same. Therefore, the BP neural network model library is established, and the corresponding network is called for operation when different patients are trained. Finally, based on sEMG signal trigger, the pneumatic glove training control algorithm was proposed. The combination of the trigger signal waveform and the motion signal waveform indicates that the pneumatic rehabilitation glove is triggered to drive the patient’s hand movement. Preliminary tests have confirmed that the accuracy rate of trend recognition for hand movement is about 90%. In the future, clinical trials of patients will be conducted to prove the effectiveness of this system.

## Introduction

Approximately two million people suffer from stroke every year in China, and about three-fourths of stroke patients have hand movement disorders (Heung et al., 2020). Moreover, the other neurological disorders, such as multiple sclerosis or motor neuron disease, also show abnormal hand movements. Patients with inflexible hands are unable to complete various actions in daily life due to lack of muscle strength and fine control of the fingers. Rehabilitation robot is playing an increasingly important role in training patients instead of rehabilitation physicians, which can improve the motor function of inflexible hands and reduce the possibility of permanent disabilities (Gaia et al., 2020; Yurkewich et al., 2020; Lemerle, Nozaki & Ohnishi, 2018). At present, the popular hand rehabilitation robots at present can be divided into finger exoskeleton rehabilitation robot (Agarwal et al., 2015; Nycz et al., 2016), flexible rehabilitation robot gloves (FRRG) and end traction finger rehabilitation robot (Bentzvi & Ma, 2014; Wu et al., 2010). Compared with other types of hand rehabilitation robots, FRRG has some advantages, including good flexibility, small size, large working space, light weight, safety and reliability (Heung et al., 2019; Mahdi, Charu & Muthu, 2019; Matthew, Jeong & Raye, 2019). Polygerinos et al. (2015) developed the rehabilitation gloves, which include a molded elastomer chamber and a fiber reinforcement that produces specific bending, twisting and extending trajectories under fluid pressure to match and support the different ranges of motion of a single finger. Wang, et al. proposed a pair of antagonistic pneumatic muscles which are very similar in action to human muscles, can be used for hand passive training (Wang et al., 2020a; Wang et al., 2020b). A new kind soft pneumatic glove with five segmented PneuNets bending actuators is made of elastomer, whose actuator driving the corresponding finger to bend (Wang, Fei & Pang, 2019). A new portable and inexpensive pneumatic rehabilitation glove is proposed in this paper.

Rehabilitation training, which is based on limb movement trend of patients, can improve the efficiency of recover (Pichiorri et al., 2015). The methods for trend recognition of human limb movement include biomechanical signal (Sangwoo et al., 2018) and bioelectrical signal (Leonardis et al., 2015). However, due to the structure and wearing characteristics of FRRG, it is expensive to install biomechanical sensors on the gloves, which make it difficult to use for patients with financial problems in their families. For patients with finger dysfunction caused by stroke, biomechanical sensors are not suitable for them and not easy to collect the biomechanical signals of their hands (Leonardo et al., 2018). On the contrary, bioelectrical signals are generated before movement, and the corresponding relationship between signals and movement can be obtained by collecting and decoding bioelectrical signals of human body, which provides an extremely important means for the prediction of human limb movement trend. There are many mature methods of limb movement intention recognition based on bioelectrical signals, including electrocorticogram (ECoG), electroencephalogram (EEG), magnetoencephalo-graphy (MEG) and electromyography (EMG). Due to the high cost of collecting ECoG, EEG or MEG signals, EMG is chosen as the bioelectrical signal for hand movement trend recognition in this paper.

EMG signals can be divided into two types; surface electromyography (sEMG) and needle in electromyography (nEMG). Compared with nEMG, sEMG has the advantages of noninvasive and simple operation. The signal collected by sEMG sensor is the sum of the potential generated by muscle activity in the area where the electrode is located on the skin surface. Selecting the appropriate muscle group of arm is very important and different muscle groups have different effects, which is reflected in the amplitude change of sEMG signals (Dai & Hu, 2020). The larger the amplitude change, the more conducive to the identification of hand movement trend. The control based on bioelectrical signal from patient muscle, mainly includes sEMG trigger control (Meng et al., 2014) and sEMG continuous control (Song et al., 2008). In this paper, a new pneumatic glove trigger control system for paralysis patients’ hand is developed. The trigger control is used to identify the movement trend of the patients, and then the assisting to complete the rehabilitation training is realized.

### Construction of pneumatic rehabilitation glove trigger control system based on sEMG

**Figure 1:**
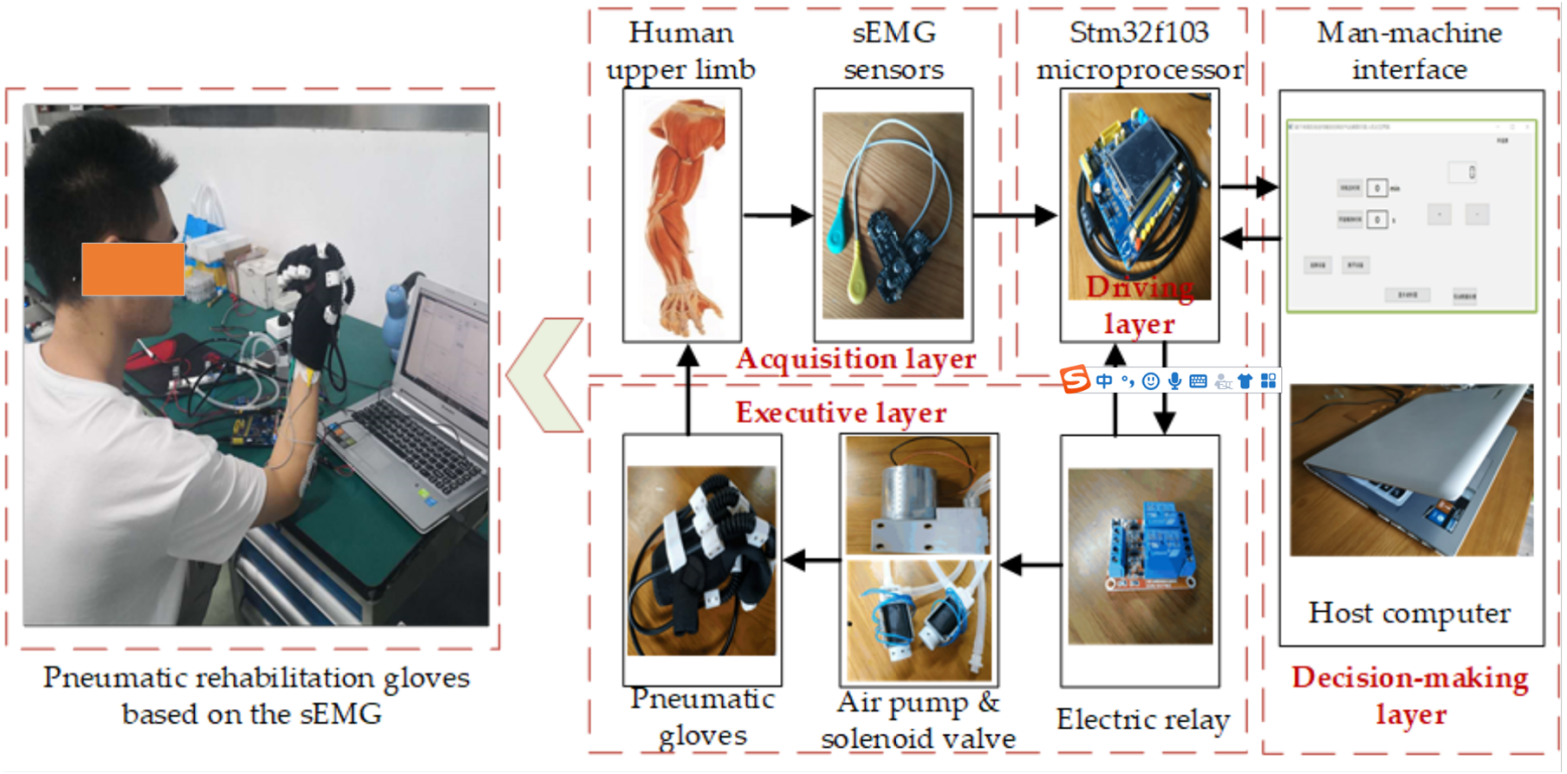
The composition of the trigger control system of the pneumatic rehabilitation gloves based on the sEMG.

The pneumatic rehabilitation glove trigger control system based on sEMG consists of one pneumatic gloves, an air pump, a Stm32f103 microprocessor equipped with an ARM chip, two electric relays, a Myoware sEMG sensor, two-position three-way solenoid valves and a host computer as shown in Fig. 1. The pneumatic rehabilitation gloves can well wrap the patients’ fingers, palms and hand back. Air pump provides power for pneumatic gloves. sEMG sensors are used to collect patient’s sEMG signals. The Stm32f103 microprocessor equipped with an ARM chip is used to process the original sEMG signals collected by sEMG sensors. It is also used as the driver of air pump and transmits the processed sEMG signals to the host computer. The host computer is developed with QT software (Cross-platform software development framework for the development of apps and devices, developed by QT Group) as the development environment. It judges the movement trend of the hand by analyzing the collected sEMG signals. According to the movement trend of the hand, it also sends related instructions to the air pump driver. Then the air pump driver controls pneumatic rehabilitation gloves to flex and extend. The above hardware platform can be divided into an acquisition layer, a decision-making layer, a driving layer and an execution layer as shown in Fig. 1. The RS232-USB (RS232 to USB) serial port is adopted between the acquisition layer and the decision layer, the decision-making layer and the drive layer. The high and low level control of the IO port pins is used between the drive layer and the execution layer. The host computer uses the QSerialPort component (Function pack of QT) to receive the sEMG signals through the RS232-USB serial port, and stores the received sEMG data in an Excel table to facilitate the subsequent static data processing.

### Processing and selection of optimal eigenvalues of sEMG signals

#### Acquisition and processing of the sEMG Signals

**Figure 2:**
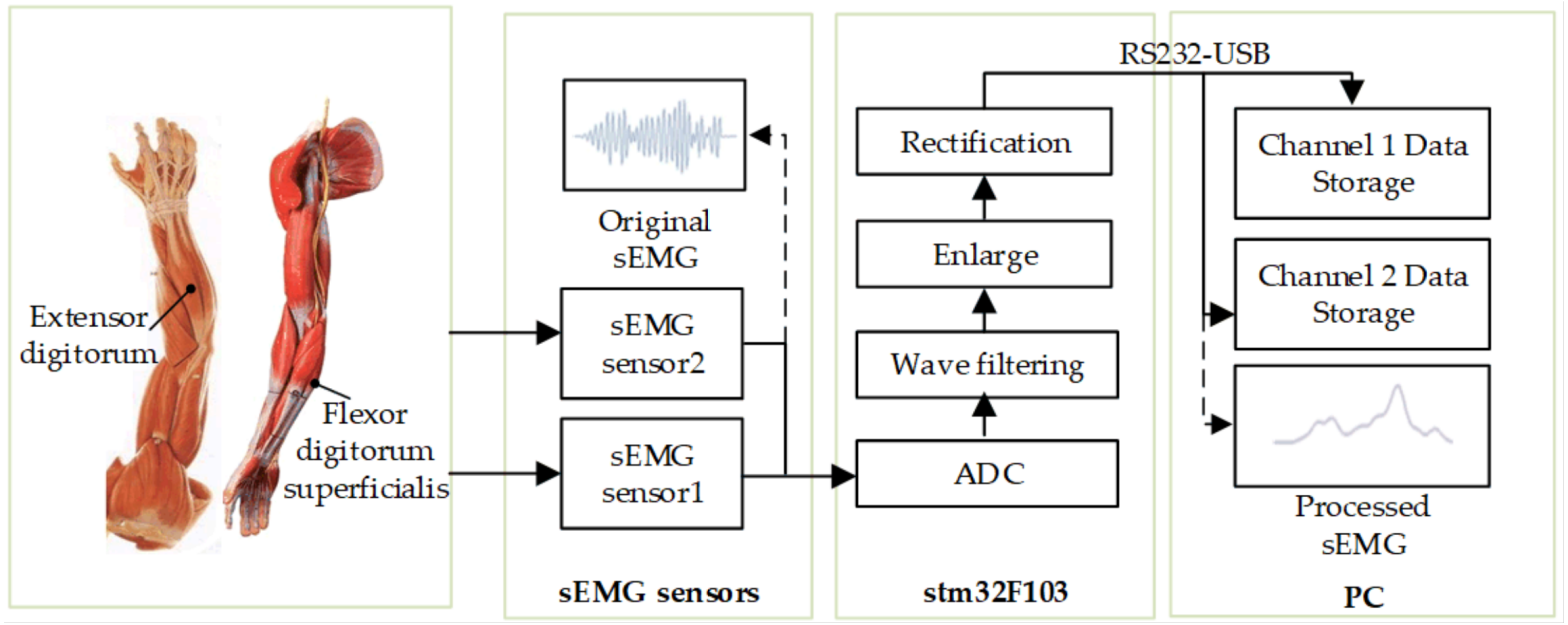
Flowchart of original sEMG signal acquisition and processing.

In order to facilitate the collection of sEMG signals, the muscle group on the forearm is selected as the collection object. The muscle groups of the forearm mainly include palmar longus, flexor carpi radialis, brachioradialis, teres pronatorus, extensor carpi radialis longus, extensor digitorum and flexor digitorum superficialis. The flexor carpi radialis is a flexor wrist muscle located on the inner side of the forearm. It starts from the medial epicondyle of the humerus and the olecranon, and ends at the proximal end of the second metacarpal bone. The flexor superficialis is mainly responsible for flexing the metacarpophalangeal joint and proximal interphalangeal joint of the 2nd to 5th fingers. The extensor digitorum can extend the metacarpophalangeal joint of the four fingers. The original sEMG signals are collected by dual-channel sEMG sensors. Each sEMG sensor has two detection electrodes and one reference electrode. The detection electrode is attached to the central part of the muscle belly of the target muscle, and the reference electrode is attached to the muscle not participating in the test exercise. The processed sEMG signal amplitude varies from 0 to 3.3V and the original sEMG signal acquisition and processing process is shown in Fig. 2.

**Figure 3:**
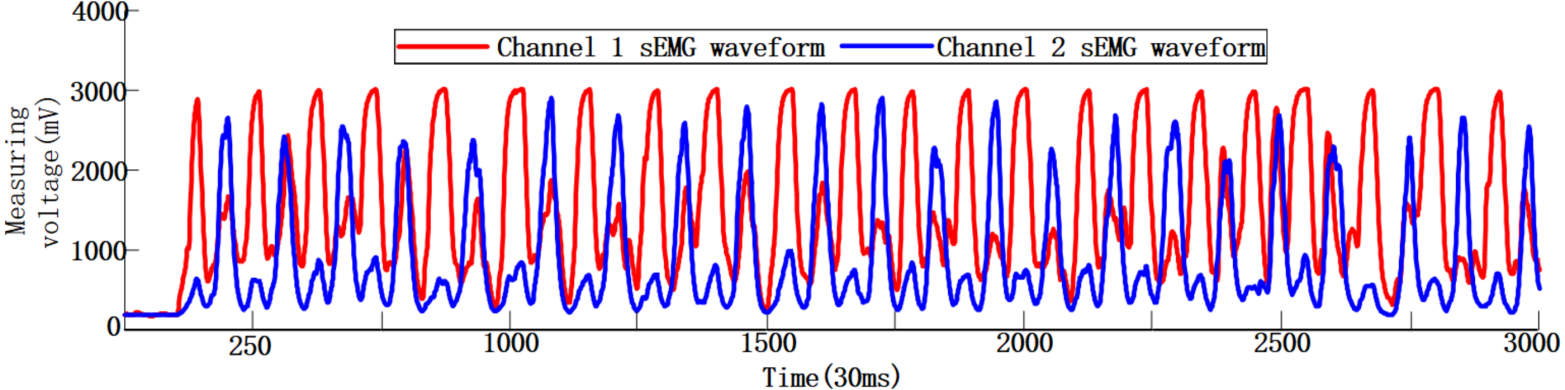
Dual-channel human hand motion sEMG waveforms in 90s.

Three healthy volunteers were recruited in this experiment with the informed consents of all volunteers and the Ethical Approval (No. [2020]LLSP(12), Ethics Committee of Faculty of Mechanical Engineering & Mechanics, Ningbo University). Volunteer 1: Male, weight 64 kg, height 175 cm, 24 years old; Volunteer 2: Male, weight 73 kg, height 177 cm, 26 years old; Volunteer 3: Male, weight 75 kg, height 180 cm, 20 years old. Using sEMG sensors and Stm32f103 microprocessor, the original sEMG signals are digitally filtered, amplified, rectified and smoothed (Lyu et al., 2020; Shi et al., 2020). After repeated experiments and comparing the amplitudes of the sEMG signals of different muscle groups collected during the same hand action, the extensor digitorum and flexor digitorum superficialis are finally selected as the muscle groups for sEMG signal collection. Volunteer 1 uses dual-channel sEMG sensors to collect the actual sEMG signals during the flexion and extension movement of his hand, as shown in Fig. 3. The total signal collection duration is about 90 s, of which the sEMG signal curves do not fluctuate much in the first 3 s, as the volunteer is in a state of inactivity. During the movement of the subject’s hand, the corresponding to the hand sEMG signal curves have changed, and the waveform in the figure appears to be convex. By observing the sEMG signals of the two channels, it can be seen that the signals of the two channels fluctuate synchronously when the subject hand is moving, but there are certain differences in the waveforms of each channel.

#### Selection of optimal eigenvalues of the sEMG signals

**Figure 4:**
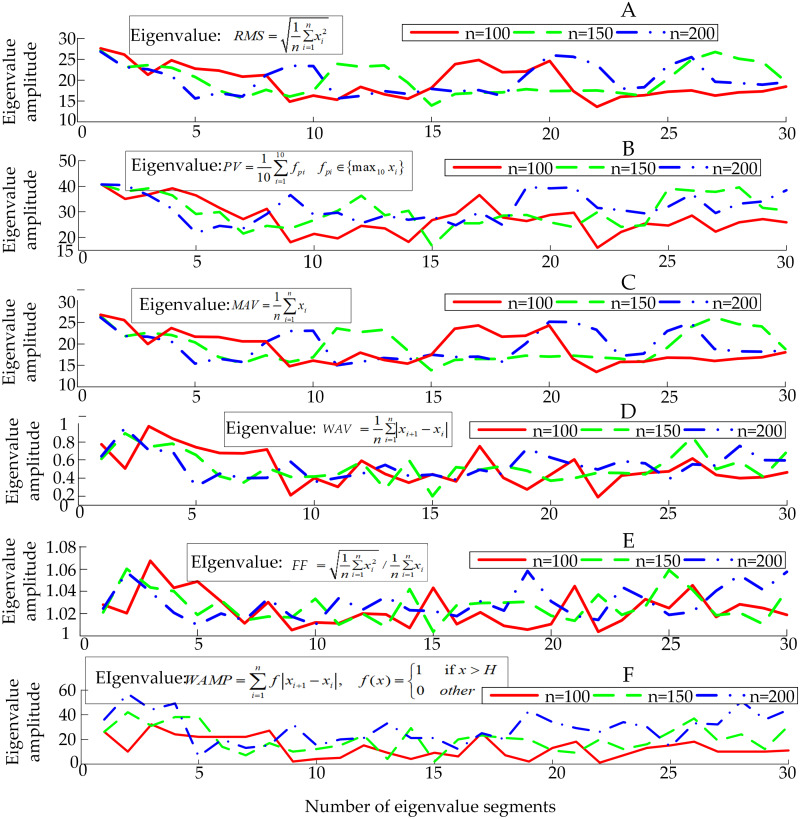
Sample of sEMG eigenvalues.

Figure 4 shows the obtained eigenvalues of sEMG sensor’s channel 1. It is necessary to use the law of statistics to find the accurate physical quantities that best represent the essence of the surface EMG signal, that is, the extracting eigenvalues of sEMG signals. The original sEMG signal after amplification, rectification and rectification integration loses a lot of frequency domain characteristics of the original signal. By directly analyzing and processing the sEMG signal in the time domain, it will be intuitive and accurate. In the time domain, the sEMG signal can be approximated as a Gaussian distribution. At present, the most commonly used time domain eigenvalues of the signal are the root mean square value (*RMS*), peak value (*PV*), mean value (*MAV*), wavelength average (*WAV*), form factor (*FF*) and Willison amplitude (*WAMP*) (Liu & Cheng, 2018). The number of eigenvalues selected is positively correlated with the accuracy of the information representation contained in the sEMG signals, but too many eigenvalues will affect the speed of the computer to make decisions, which is manifested in the deterioration of the follow ability of the pneumatic gloves to the patient’s intention. On the contrary, if the selected number of eigenvalues of the sEMG signal is too small, the pneumatic rehabilitation glove control system cannot accurately recognize the patient’s movement intention. *x*_*i*_ represents the amplitude of the signal, and *n* represents the extracted step size. First, *N* (*N* = 30) groups of sEMG signals are extracted to form sEMG samples with empirical steps *n* = 100, *n* = 150, *n* = 200 in the continuously collected sEMG signals respectively as W1, W2, and W3. And then the above-mentioned 6 eigenvalues with each segment length as the unit to form an eigenvalue sample *E*_*i*_ (6 ×*N*) is calculated, where *i* = 1, 2, 3 corresponds to the sEMG samples W1, W2, W3, respectively.

The patient’s hand movement trend will be expressed as fluctuations in sEMG signals. The eigenvalues of the signals reflect the nature of the signals over a period of time, so the fluctuation of the sEMG will also be specifically reflected in the fluctuation of sEMG eigen-values. According to prior knowledge, it can be known that the greater the degree of dispersion of eigenvalues, the more conducive the neural network to the recognition of the movement trend based on eigenvalues. Based on the six eigenvalues, three eigenvalues with a large degree of dispersion will be selected as the parameters of the next action classification, participating in the training and testing of the neural network for intention recognition. Since a single dispersion index is not sufficient to fully characterize the degree of dispersion of the signals, 4 dispersion indicators will be used to process the 6 eigenvalues that have been obtained, namely range (*R*), interquartile range (*Q*), and variance (*V*) and fourth-order center distance (*K*).

Range is the difference between the maximum and minimum values between data. The greater the range, the greater the degree of dispersion, namely:

(1) \begin{eqnarray*}R=\max \left( {s}_{i} \right) -\min \left( {s}_{i} \right) .\end{eqnarray*}

The interquartile range represents the range of the middle half of the data. The larger the interval, the greater the degree of dispersion. Arrange a set of data in ascending order. The number in the *x%* position is represented by *P*_*x*_. The lower quartile and upper quartile are *P*_8_ and *P*_23_ respectively, namely:

(2) \begin{eqnarray*}Q={P}_{23}-{P}_{8}.\end{eqnarray*}

Variance describes the degree of dispersion of data mathematical expectation, that is, the greater the variance, the greater the degree of dispersion, namely:

(3) \begin{eqnarray*}V= \frac{1}{N} \sum _{i=1}^{N}{ \left( {s}_{i}- \frac{1}{N} \sum _{i=1}^{N}{s}_{i} \right) }^{2}.\end{eqnarray*}

The fourth-order center distance is a cumulative numerical statistics reflecting the distribution characteristics of random variables. The larger the fourth-order center distance, the smaller the degree of dispersion, namely:

(4) \begin{eqnarray*}K= \frac{ \frac{1}{N} \sum _{i=1}^{N}{ \left( \left\vert {s}_{i} \right\vert - \frac{1}{N} \sum _{i=1}^{N}{s}_{i} \right) }^{4}}{{ \left( \frac{1}{N} \sum _{i=1}^{N}{s}_{i}^{2} \right) }^{2}} .\end{eqnarray*}

**Figure 5:**
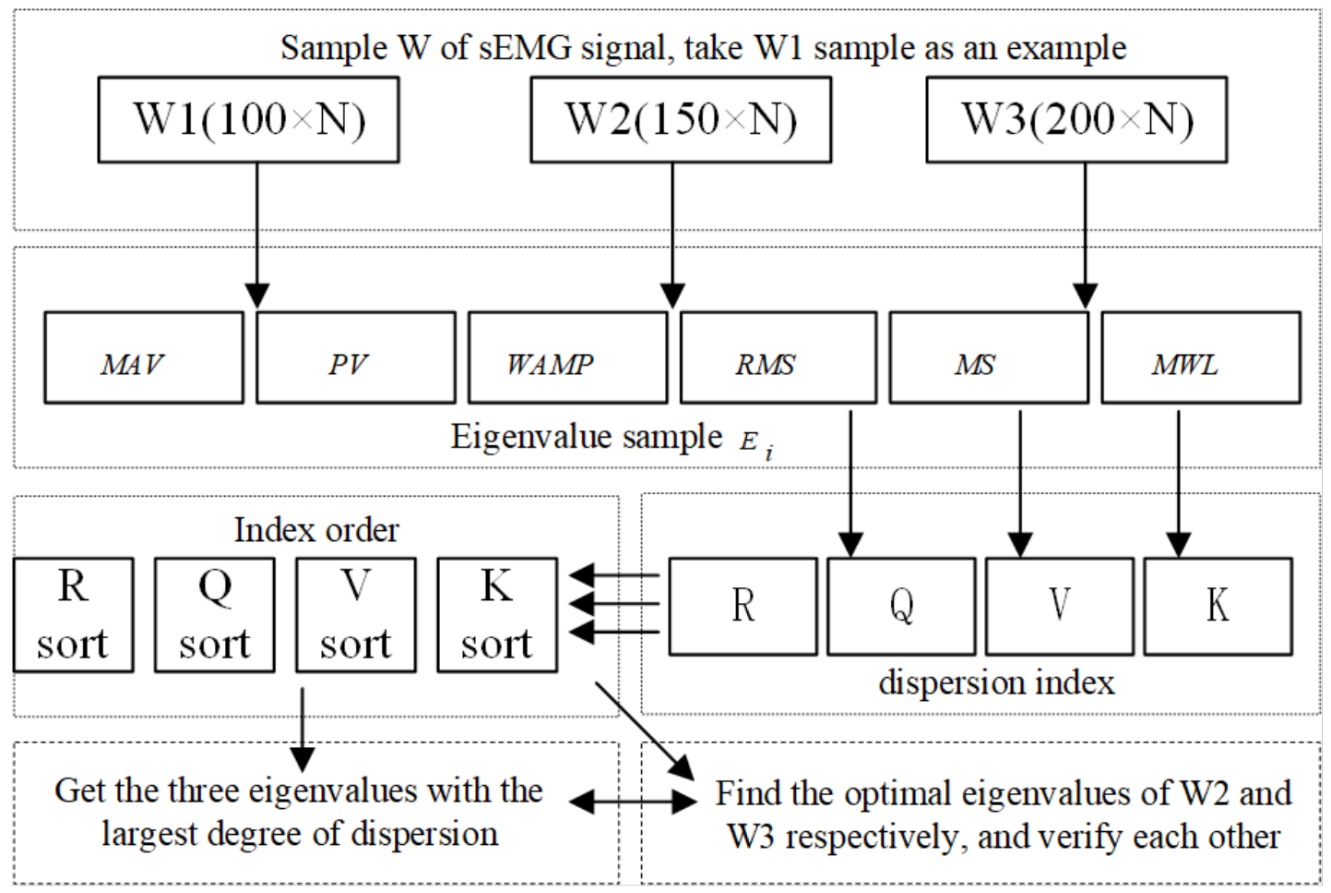
The flow chart of finding the optimal eigenvalues.

In Eqs. (1) and (4), *S*_*i*_ represents the data amplitude and *N* represents the data length. The process of determining the optimal eigenvalue is shown in Fig. 5.

By observing the sorting results of the data dispersion degree in Table 1, three eigenvalues with the largest dispersion degree are selected, which are *WAMP*, *PV* and *RMS*. For further verification, the dispersion index of *E*_2_ and *E*_3_ are calculated by the same method, and a comprehensive ranking is performed according to the magnitude of the dispersion index, as shown in Tables 2 and 3.

### Research on hand movement trend recognition based on BP neural network

Using the collected sEMG signals to achieve the purpose of identifying the patient’s finger movement trend is the main problem in the design of the pattern recognition classifier. The back propagation (BP) neural network model was chosen to construct the motion recognition classifier, as the BP neural network model has good self-learning, nonlinear mapping and adaptation, generalization and fault tolerance (Wang et al., 2020a; Wang et al., 2020b). It could be an ideal movement trend pattern recognition tool.

**Table 1:** *E*_1_ dispersion index magnitude ordering.

**Valuesindex**	***MAV***	***PV***	***RMS***	***WAMP***	***FF***	***WAV***	**Dispersion index order**
***V***	12.1356	33.2040	12.6915	66.0600	0.0003	0.0283	4, 2, 3, 1, 6, 5
***R***	13.3068	24.6861	14.0063	31.0000	0.0781	0.7957	4, 2, 3, 1, 6, 5
***Q***	1.0042	3.0947	1.3145	5.0000	0.0070	0.1064	4, 2, 3, 1, 6, 5
***K***	0.0020	0.0034	0.0019	0.1177	0.0002	0.0189	3, 4, 2, 6, 1, 5

**Table 2:** *E*_2_ dispersion index magnitude ordering.

**Valuesindex**	***MAV***	***PV***	***RMS***	***WAMP***	***FF***	***WAV***	**Dispersion index order**
***V***	10.8368	30.9901	11.1964	108.8024	0.0002	0.0216	4, 2, 3, 1, 6, 5
***R***	12.6620	24.0479	13.1707	40.0000	0.0578	0.7017	4, 2, 3, 1, 6, 5
***Q***	1.0578	5.4918	1.3927	10.0000	0.0078	0.0910	4, 2, 3, 1, 6, 5
***K***	0.0016	0.0024	0.0014	0.0705	0.0003	0.0134	3, 4, 2, 6, 1, 5

**Table 3:** *E*_3_ dispersion index magnitude ordering.

**Valuesindex**	***MAV***	***PV***	***RMS***	***WAMP***	***FF***	***WAV***	**Dispersion index order**
***V***	10.6075	27.7494	11.0703	164.1194	0.0002	0.0191	4, 2, 3, 1, 6, 5
***R***	11.1623	18.9855	11.2990	51.0000	0.0487	0.6539	4, 2, 3, 1, 6, 5
***Q***	3.4440	4.6856	4.1505	16.0000	0.0130	0.1500	4, 2, 3, 1, 6, 5
***W***	0.0014	0.0015	0.0013	0.0524	0.0004	0.0121	3, 4, 2, 6, 1, 5

### Construction of BP neural network classifier

**Figure 6:**
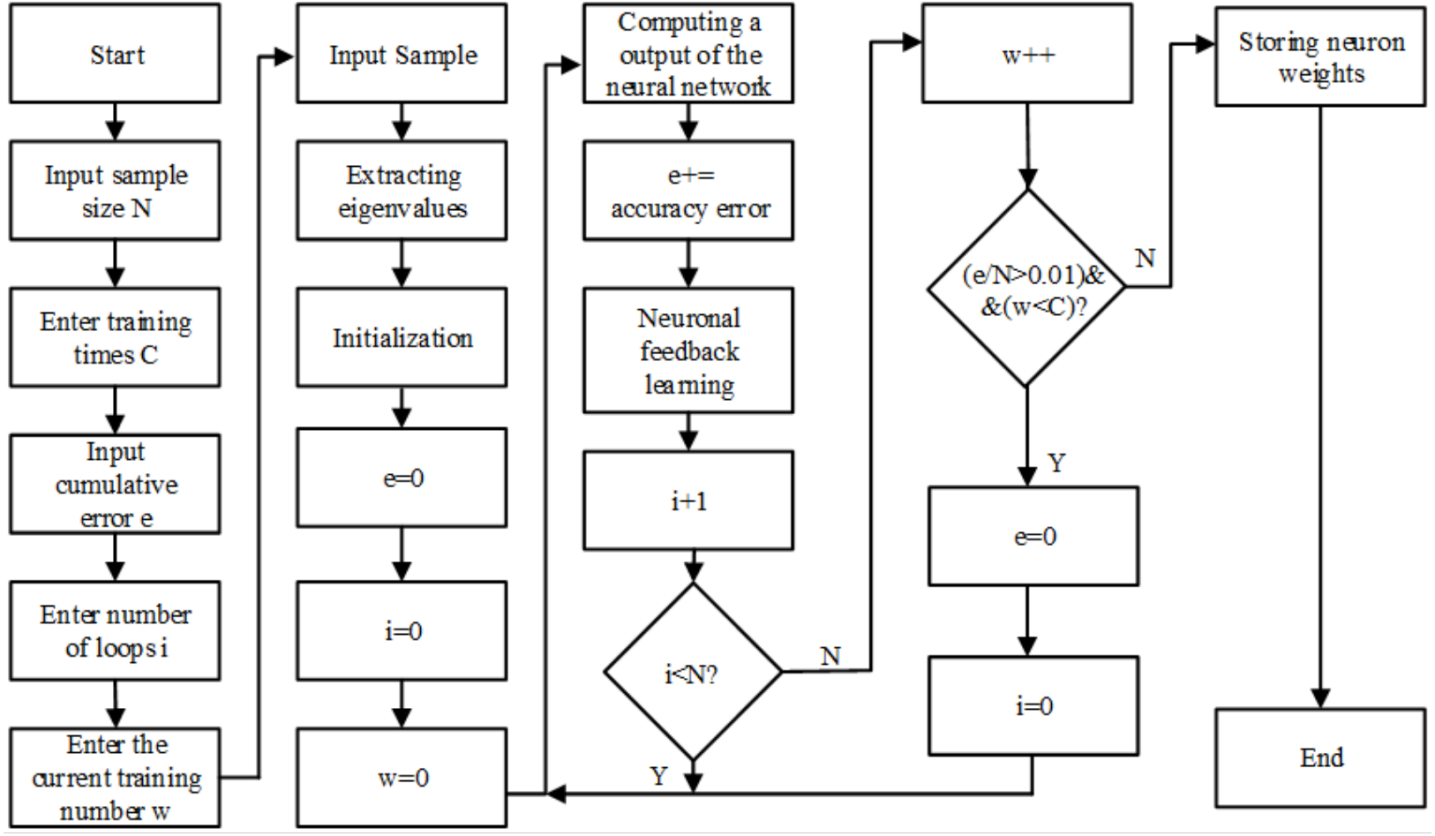
Flowchart of training algorithm for BP network.

BP neural network is an adaptive nonlinear dynamic system composed of a large number of interconnected neurons. It can learn and store the mapping relationship of multiple input–output modes without describing specific mathematical equations in advance. The quality of neural network classifiers is closely related to the number of neural network layers, the number of nodes in each layer, the transfer function of the hidden layer, and the learning algorithm. The training algorithm flow chart of constructing BP neural network under QT software development environment is shown in Fig. 6.

The number of BP neural layers is selected as 3 layers, namely, the input layer (I), the hidden layer (H) and the output layer (O). This is because Robert Hecht-Nielson proved that a three-layer neural network can complete the mapping of any n-dimensional input and m-dimensional output, so in order to simplify the calculation, a three-layer network is adapted (Hecht-Nielsen, 1992).

Hidden layer transfer function:

(5) \begin{eqnarray*}{y}_{i}= \frac{({x}_{i}-MinValue+A)}{MaxValue-MinValue+A} \end{eqnarray*}

Transfer function of the output layer:

(6) \begin{eqnarray*}{y}_{k}={x}_{k}\times (MaxValue-MinValue+A)-A+MinValue\end{eqnarray*}

Logsig activation function is used:

(7) \begin{eqnarray*}{y}_{j}= \frac{1}{1+{e}^{-{x}_{i}}} \end{eqnarray*}

Levenberg-Marquart (L-M) learning algorithm is used:

(8) \begin{eqnarray*}\Delta \omega ={ \left( {J}^{T}J+\mu I \right) }^{-1}g{J}^{T}e\end{eqnarray*}

In Eqs. (5) and (6), *MinValue* is the minimum value of the input layer value; *MaxValue* is the maximum value of the input layer value; constant *A* reprevents the denominator from being zero; *x*_*i*_ represents the eigenvalue extracted from the sEMG signals; *y*_*i*_ represents the normalized feature value of the input layer; *x*_*k*_ represents the output value of the hidden layer, and *y*_*k*_ represents the final output value of the output layer. In Eq. (7), *x*_*i*_ represents the sum of the product of the output value and the weight of each neuron in the previous network, *y*_*j*_ represents the output of the *j* neuron in the current layer network. In Eq. (8), *J* represents the Jacobian matrix of the derivative of weights from network error, *e* represents the error vector, µisan adaptive constant, which is greater than 0. The input layer is a 6 ×1 vector composed of the optimal eigenvalues of the 2-channel sEMG signals, so the number of nodes in the input layer is 6, set the number of nodes in the output layer to 1, and use the output result of the output layer to determine the triggered action. The action code is built as in Table 4.

**Table 4:** Action coding.

Action type	Action encoding
Action	1
No action	0

The number of hidden layer nodes is determined by the following empirical formula (Sheela & Deepa, 2013):

(9) \begin{eqnarray*}{n}_{1}=\sqrt{n+m}+a\end{eqnarray*}

where, *n* is the number of input nodes; *m* is the number of output nodes; *n*_1_ is the number of hidden nodes; *a* is a constant between 1 and 10.

The number of hidden nodes gradually increases, and the training error of the neural network is observed during this process. As the number of hidden layer nodes increases, the training error gradually decreases, but after a certain number of nodes, the test error will fluctuate greatly. Therefore, considering the trend of training and test error changes, the number of hidden layer nodes is finally determined to be 12.

### Training and testing of BP neural network

In order to realize the mapping function of the input matrix and the output matrix, the BP neural network needs to be trained. The feedback mechanism of BP neural network includes two parts. One is that the BP neural network produces prediction results. The other is to compare the prediction results with sample results, and then correct the neuron error until the error meets the specified requirements or reaches the specified number of training sessions. 160 sets of data are used as training samples to train the BP neural network as shown in Table 5. Each set of data contains the input and target output of the BP neural network. The input is the optimal eigenvalues of the sEMG signals collected by the two channels of the sEMG sensors, and the output is the code value of the corresponding action.

**Table 5:** Part training sample data.

**Channel**	**Eigenvalue**	**Sample1**	**Sample 2**	**Sample 3**	**Sample 4**	**Sample 5**
**Channel 1**	*WAMP*	32	0	24	5	22
	*RMS*	21.3020	10.4378	24.7514	19.4136	22.7687
	*PV*	36.8406	11.8223	39.2490	29.2650	36.4782
**Channel 2**	*WAMP*	45	0	46	0	32
	*RMS*	26.6754	16.1020	26.8007	17.8136	24.4910
	*PV*	41.85703	16.8022	41.1995	20.2604	37.9465
	Action encoding	1	0	1	0	1

Before training the BP neural network, the training samples need to be randomly divided into two types at a ratio of 3:1, as training samples and test samples separately. After the BP neural network uses the training sample to complete each iteration, it is judged whether the average error value meets the accuracy requirements (*e* < 0.01). If the accuracy requirements are met, the training is completed. Otherwise, the prediction results are compared with the sample target results, and then start neural Meta-feedback learning, repeat the above steps until reaching the specified number of training times or meet the accuracy requirements to complete the training.

**Figure 7:**
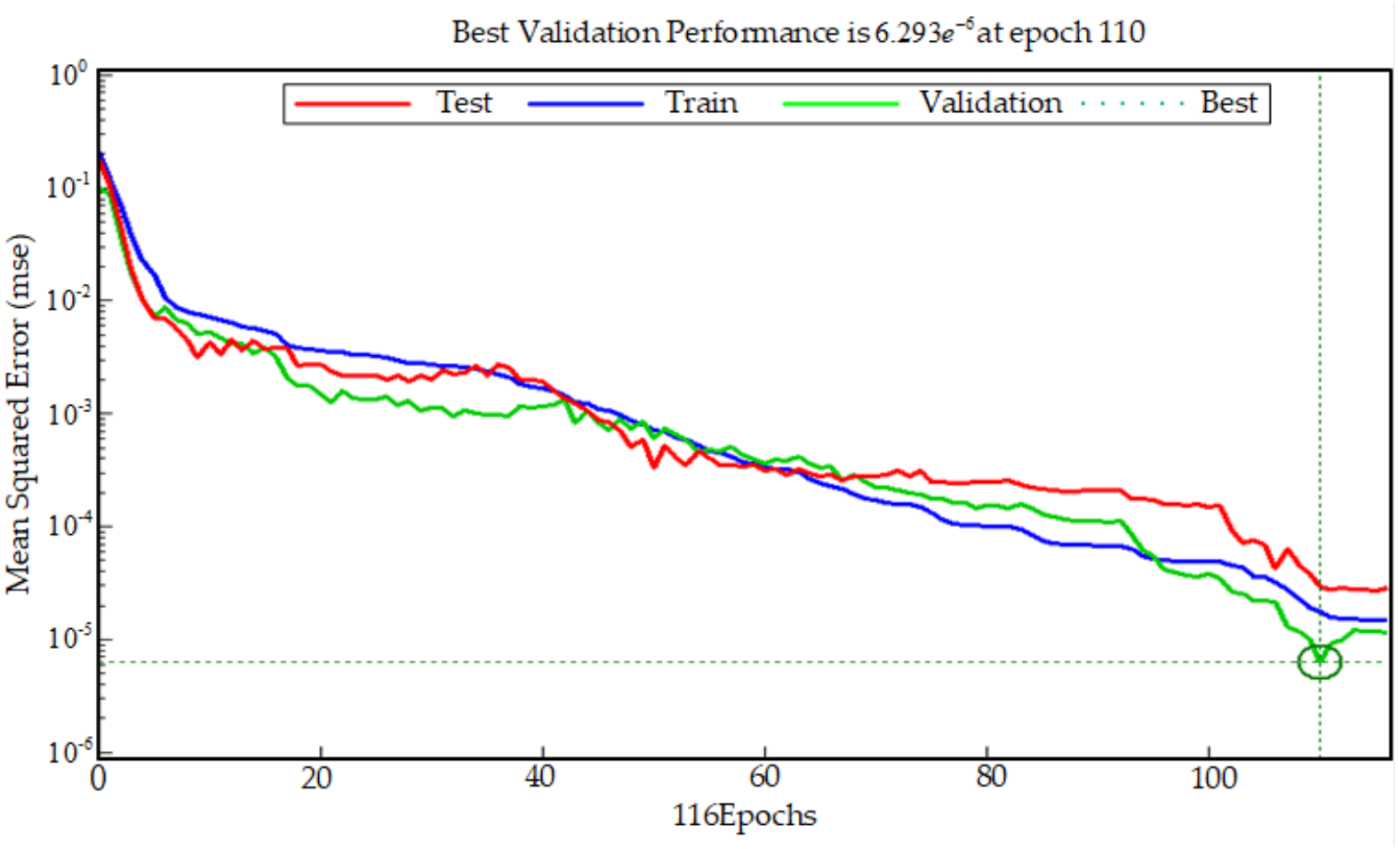
Best validation performance.

**Figure 8:**
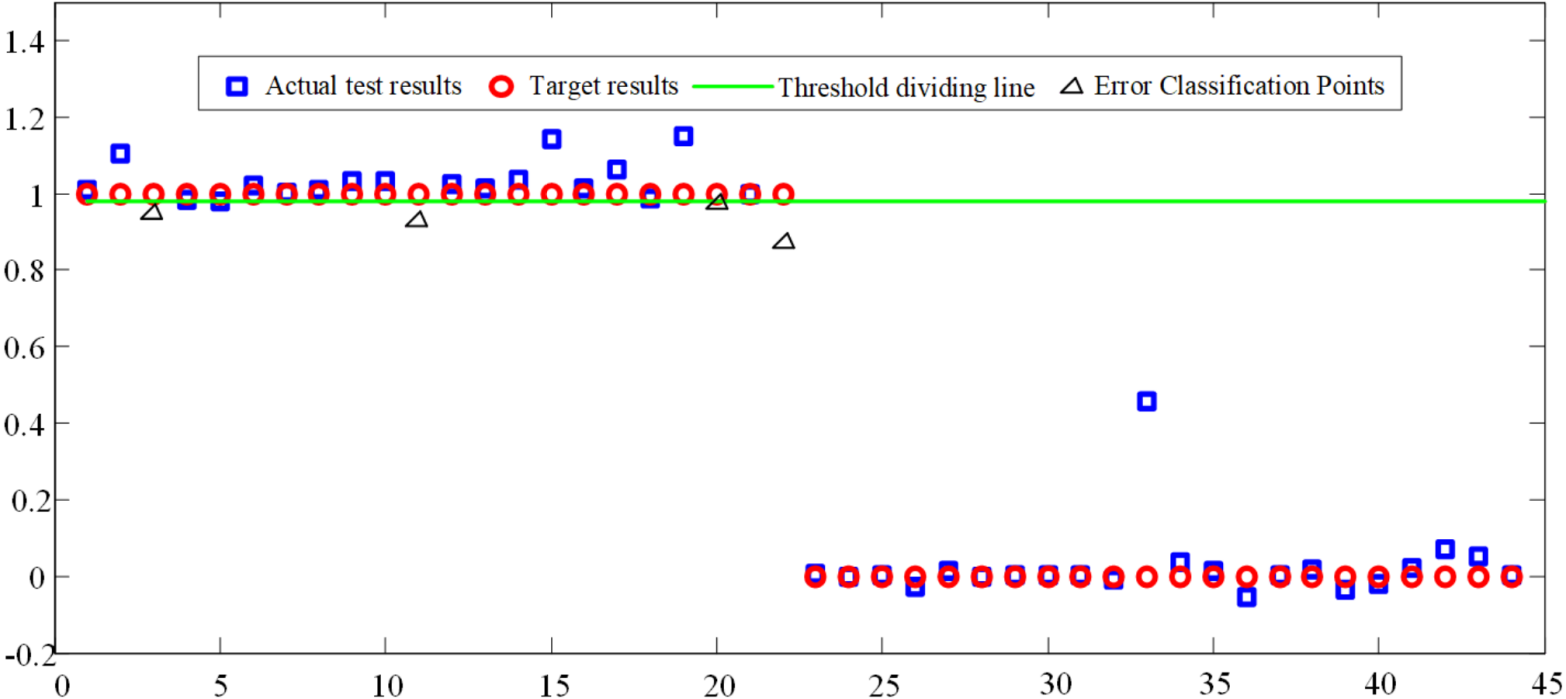
BP network motion classification results.

Considering that BP neural network is prone to over training and lack of generalization ability, the training samples input into the neural network training algorithm are divided into three kinds of samples: train samples, validation samples and test samples. In each epoch of training, the errors between the results of three samples and the target results are tested. When the error of validation samples does not decrease in six successive epochs, the training of BP neural network is stopped to prevent over fitting, which is caused by overtraining of BP neural network. It can be seen from Fig. 7 that the total number of epochs of BP neural network is 116. After 110 epoch of BP neural network, the error of train samples, the error of test samples and the error of validation samples no longer have a downward trend, or their downward trend is not obvious. The best validation performance is 6.293e^−6^. Therefore, the training of BP neural network is finished at the 116th epoch. The threshold *w* is set 0.98, and the trained BP neural network is used to classify and recognize patient actions, the recognition result is shown in Fig. 8. Common classification performance measures are Precision (*PRE*), Recall (*REC*), and the harmonized average of the two (*F*_1_).

According to Table 6, the calculation formula of *Pre*, *Rec* and *F*_1_**:**

(10) \begin{eqnarray*}Pre= \frac{TP}{TP\mathit{ + }FP} \end{eqnarray*}

(11) \begin{eqnarray*}Rec= \frac{TP}{TP\mathit{ + }FN} \end{eqnarray*}

(12) \begin{eqnarray*}{F}_{1}=2 \frac{PR\cdot REC}{PR+REC} \end{eqnarray*}

From Eqs. (10)–(12), *Pre* = 1, *Rec* = 0.818, *F*_1_ = 0.8998.

### Active trigger control strategy for pneumatic gloves

**Figure 9:**
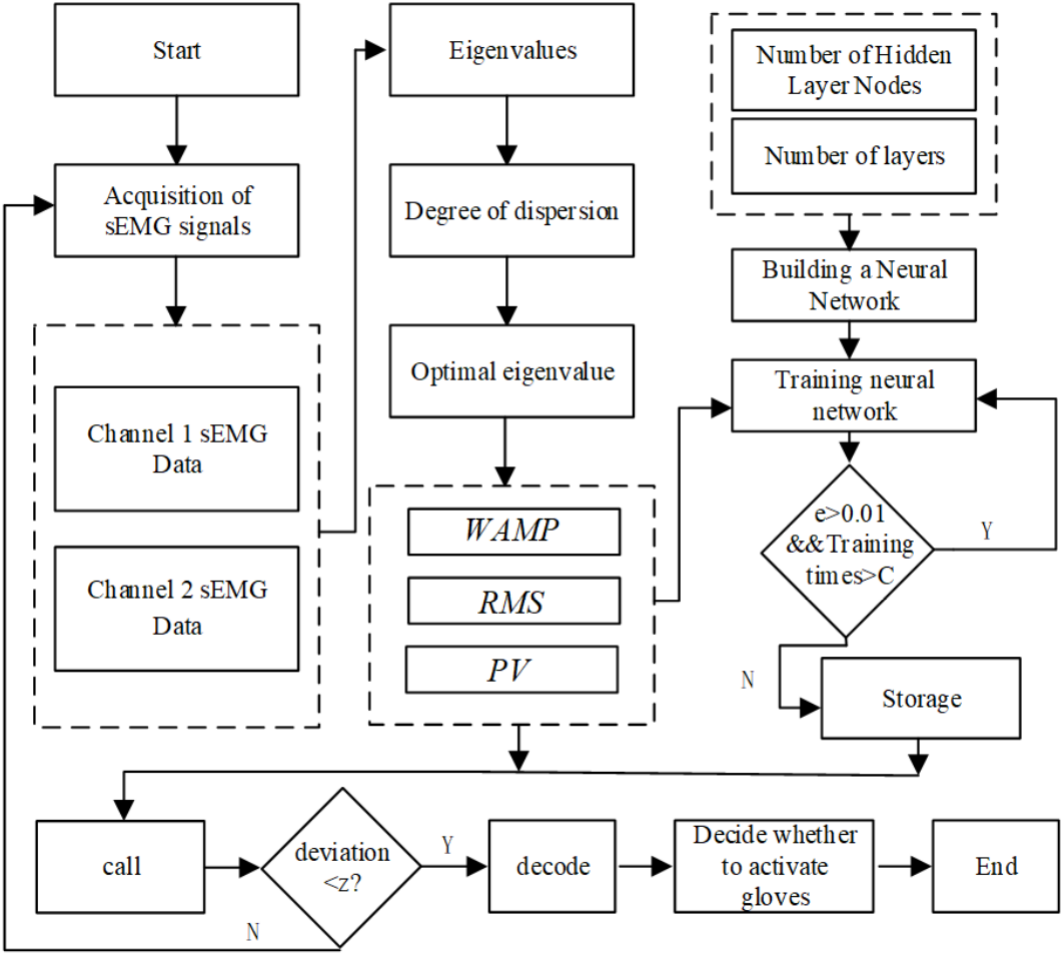
Algorithm flow chart of pneumatic glove trigger based on sEMG signals.

The software processing algorithm of the control system mainly includes a two-channel optimal eigenvalue amplitude calculation and a BP neural network action recognition calculation. Among them, the same optimal eigenvalue is selected for different patients, and the eigenvalue amplitude calculation formula is unique. However, due to differences between individuals, the weights and thresholds of the nodes in the BP neural network model corresponding to different patients are not the same, so the BP neural network model library needs to be established in the actual application process. Different patients call their corresponding BP neural network models during training. When a patient conducts active training based on sEMG signals for the first time, he needs to collect sEMG signals under the guidance of a physician, and complete the training of the BP neural network, and store the required neural network in the BP neural network model library. The corresponding database will be called during a training session. The algorithm flow of active trigger control strategy for pneumatic rehabilitation gloves based on sEMG signals is shown in Fig. 9.

**Table 6:** Part training sample data.

**Predectionresult** / **real Result**	***Positive(+)***	***Negative(-)***	***Total***
***Positive(+)***	18 (TP)	4 (FN)	22 (TP+FN)
***Negative(-)***	0 (FP)	22 (TN)	22 (FP+TN)
***Total***	18 (TP+FP)	26 (FN+TN)	

## Results

**Figure 10:**
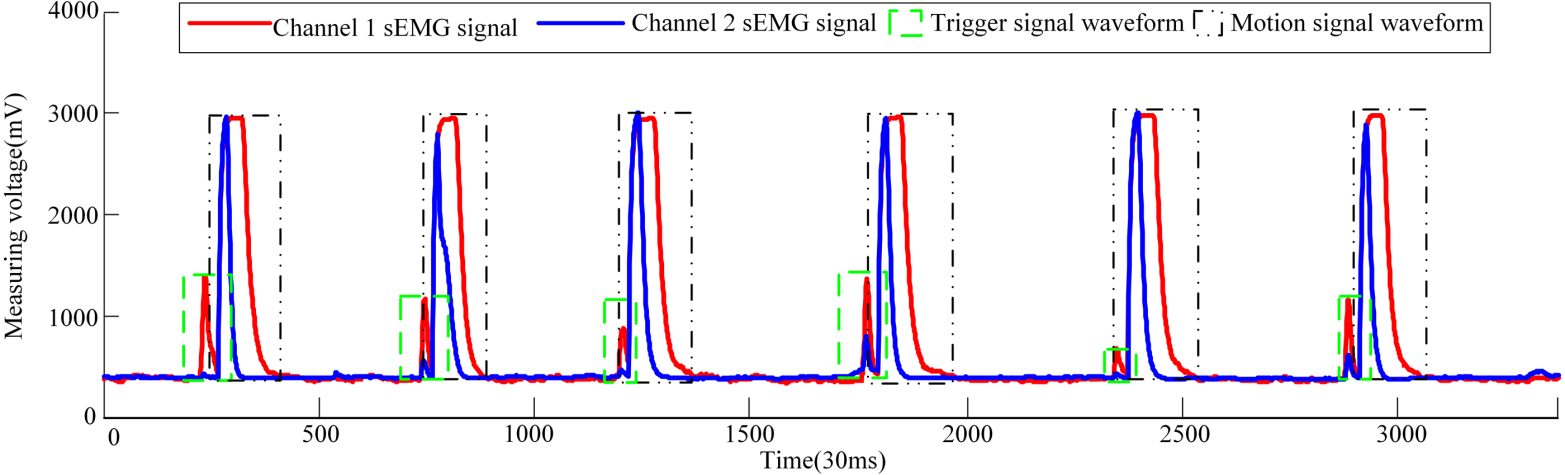
Volunteer 1′s sEMG signal waveform when he attended pneumatic rehabilitation gloves triggering control.

**Figure 11:**
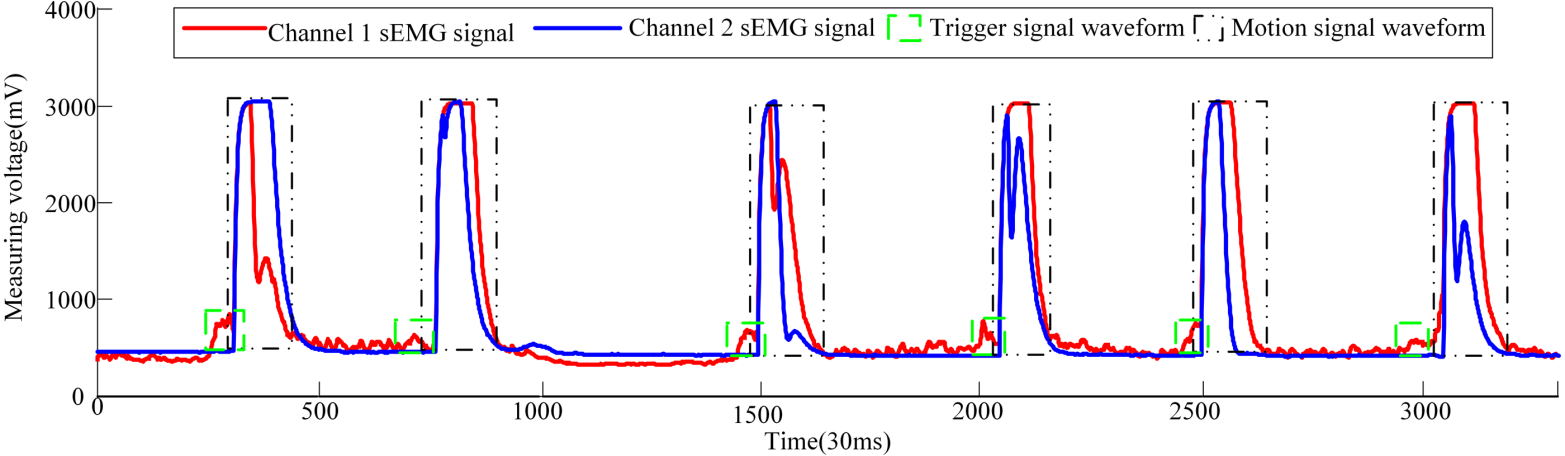
Volunteer 2′s sEMG signal waveform when he attended pneumatic rehabilitation gloves triggering control.

**Figure 12:**
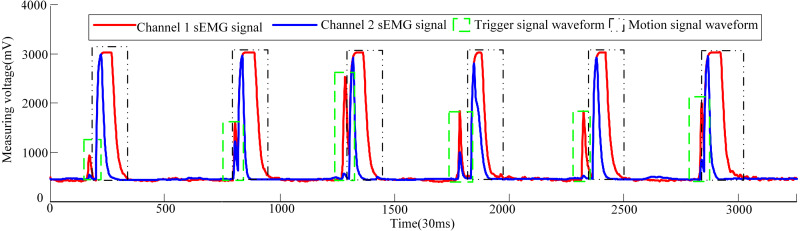
Waveforms of dual-channel sEMG signals when three volunteers attend pneumatic rehabilitation gloves triggering control.

Now three male volunteers apply the above sEMG signal control strategy to identify the volunteer’s hand movement trend to trigger the pneumatic rehabilitation gloves. Three volunteers are required to complete the triggering of the pneumatic rehabilitation gloves six times within 100s, and the time from triggering to the completion of the training of a single pneumatic rehabilitation gloves should exceed 10s. The accuracy of the control system can be checked by completing the specified number of experiments within the specified time. The time to complete a single experiment is set to exceed 10s in order to make the extracted sEMG signal more intuitive. When the three volunteers realized the trigger control of the pneumatic gloves, the waveform diagram of the sEMG signal is shown in Figs. 10, 11 and 12. The surface EMG signal waveform without fluctuation in the figures indicates that the pneumatic rehabilitation gloves have not been triggered. At this time, the output of the control algorithm is 0. However, the combination of the trigger signal waveform and the motion signal waveform indicates that the pneumatic rehabilitation gloves are triggered to drive the patient’s hand muscle movement. At this time, the output of the control algorithm is 1. All of the movement trends of the three volunteers were correctly identified, which indicates that the active triggering training based on sEMG signals may have universal applicability.

## Discussion

In order to realize active triggering training becoming possible in home rehabilitation, EMG is chosen as the bioelectrical signal for hand movement trend recognition, replacing the other high cost of collecting ECoG, EEG or MEG signals. The rehabilitation gloves’ hardware platform can be divided into an acquisition layer, a decision-making layer, a driving layer and an execution layer.

The control system uses the BP neural network as a classifier for patient’s hand movement trend recognition, and extracts the characteristic values of sEMG signals in the time domain: *MAV*, *PV*, *WAMP*, *RMS*, *MS* and *MWL*, and then through the degree of dispersion index *R*, *Q*, *V* and *K*, the optimal eigenvalues of the sEMG signals are selected. By observing the sorting results of the data dispersion degree in Table 1, three eigenvalues with the largest dispersion degree are selected, which are *WAMP*, *PV* and *RMS*. By observing Tables 2 and 3, it can be seen that the most discrete eigenvalues extracted by samples W2 and W3 are *WAMP*, *PV* and *RMS*, which are the same as the optimal eigenvalues corresponding to the W1 sample. By comparing Tables 1, 2, and 3, it can be seen that the order of the dispersion degree of each eigenvalues corresponding to different sub-samples is roughly the same. The magnitude of the dispersion index of the selected optimal eigenvalue is significantly higher than other eigenvalues. So it is reasonable to comprehensively select the optimal eigenvalues in the time domain as *WAMP*, *PV* and *RMS*.

*WAMP*, *PV* and *RMS* are used as the input values of the BP neural network. On the basis of the BP neural network which is used to establish the classifier of hand movement, the mapping relationship between hand sEMG signals and hand actions is finally completed by training and testing. From the Fig. 8, when the actual test result is greater than *w*, the test result is equal to the action target result; when the test result is less than *w*, the test result is equal to the non-action target result. The accuracy of trend recognition is determined by judging whether the test result is equal to the corresponding target test result. A total of 44 judgments are made in the Fig. 8, only 4 of which are wrong as shown by the triangle. Based on this, it can be considered that the correctness rate of BP judgment is about 90%. Based on Fig. 7, the train correctness rate of BP judgment is about 99.997%. Judging the main reason for the distortion is closely related to factors such as the quality of the electrode paste, the state of the skin on the surface of the human body, and the changes in the muscle group during the sEMG acquisition process.

The pneumatic rehabilitation glove training control algorithm, based on sEMG signal, was proposed. By observing the sEMG signal waveforms of three volunteers, it can be found that when the BP neural network monitors the hand’s movement trend, the pneumatic gloves will be triggered to drive the fingers to perform rehabilitation training. The difference in the amplitude and duration of the trigger signal of different volunteers in Figs. 10, 11 and 12 is related to the volunteer’s different physical quality, the duration and intensity of hand movement trend. Three male healthy volunteers used the control system to achieve the experimental results of the trigger experiment on pneumatic rehabilitation gloves, which preliminarily confirmed that the system has a high accuracy rate for hand movement trend recognition, and it may be useful in patient active hand training.

In the future, more healthy volunteers will be recruited to participate in this experiment. The generality and accuracy of this trigger control system for the recognition of different people’s hand movement trend are tested in a larger range. Then stroke patients will be recruited to participate in the experiment to test. Comparison between the rehabilitation effect of traditional pneumatic rehabilitation robot and the ones with the trigger control system on stroke patients will be conducted. At last, the feasibility of applying the device to finger paralysis caused by different diseases will be considered. Meanwhile, we will also consider the effects of spasm, complete plegia and other factors on the accuracy of the trigger system.

## Conclusions

An active trigger control system for pneumatic rehabilitation gloves, based on sEMG signals, is developed, which could achieve immediate rehabilitation movement trend to help the patient complete active hand rehabilitation training. Firstly, acquisition and processing of the sEMG signals from the human is researched, and three optimal eigenvalues of sEMG signals were selected as the follow-up neural network classification input. Then, based on BP neural network, the neural network classifier of hand movement is constructed. Moreover, the mapping relationship between hand sEMG signals and hand actions is built by training and testing. Based on the individual differences, the corresponding BP neural network model database of different people was established. At last, the pneumatic glove training control algorithm was proposed. Preliminary experiment shows that the combination of the trigger signal waveform and the motion signal waveform indicates that the pneumatic rehabilitation glove is triggered to drive the patient’s hand movement. The device has high accuracy rate of trend recognition for hand movement. The above research could produce important scientific value for the development of robot technology and rehabilitation theory, provide theoretical basis and technical support for the control strategy of new hand rehabilitation robots.

##  Supplemental Information

10.7717/peerj-cs.448/supp-1 Supplemental Information 1 Volunteer 1 data

10.7717/peerj-cs.448/supp-2 Supplemental Information 2 Volunteer 2 data

10.7717/peerj-cs.448/supp-3 Supplemental Information 3 Volunteer 3 data

10.7717/peerj-cs.448/supp-4 Supplemental Information 4 Fig. 1 raw data

10.7717/peerj-cs.448/supp-5 Supplemental Information 5 Training raw data

10.7717/peerj-cs.448/supp-6 Supplemental Information 6 Code

## Additional Information and Declarations

### Data Availability

The following information was supplied regarding data availability: Raw data and code are available in the Supplemental Files.

## References

[ref-1] Agarwal P, Fox J, Yun Y, O’Malley MK, Deshpande A (2015). An index finger exoskeleton with series elastic actuation for rehabilitation. International Journal of Robotics Research.

[ref-2] Bentzvi P, Ma Z (2014). Sensing and Force-Feedback Exoskeleton (SAFE) Robotic Glove. IEEE Transactions on Neural Systems and Rehabilitation Engineering.

[ref-3] Dai CY, Hu XG (2020). Finger joint angle estimation based on motoneuron discharge activities. IEEE Journal of Biomedical and Health Informatics.

[ref-4] Gaia VP, Jeanette P, Loïc C, Pauline R, Jean-Claude B, Elena P, Jörgen B, Påvel GL (2020). Recovery and prediction of dynamic precision grip force control after stroke. Stroke.

[ref-5] Hecht-Nielsen (1992). Theory of the backpropagation neural network. Neural Networks for Perception.

[ref-6] Heung HL, Tang ZQ, Shi XQ, Tong RKY, Li Z (2020). Soft rehabilitation actuator with integrated post-stroke finger spasticity evaluation. Frontiers in Bioengineering and Biotechnology.

[ref-7] Heung KHL, Tong RKY, Lau ATH, Li Z (2019). Robotic glove with soft-elastic composite actuators for assisting activities of daily living. Soft Robotics.

[ref-8] Lemerle S, Nozaki T, Ohnishi K (2018). Design and evaluation of a remote actuated finger exoskeleton using motion-copying system for tendon rehabilitation. IEEE Transactions on Industrial Informatics.

[ref-9] Leonardis D, Barsotti M, Loconsole C, Solazzi M, Troncossi M, Mazzotti C, Castelli VP, Procopio C, Lamola G, Chisari G, Bergamasco M, Frisoli A (2015). An EMG-controlled robotic hand exoskeleton for bilateral rehabilitation. IEEE Transactions on Haptics.

[ref-10] Leonardo C, Meyer JT, Galloway KC, Peisner JD, Ganberry R, Wagner DA, Sven E, Sabrina P, Walsh CJ (2018). Assisting hand function after spinal cord injury with a fabric-based soft robotic glove. Journal of Neuroengineering and Rehabilitation.

[ref-11] Liu Y, Cheng L (2018). Spiking-neural-network based Fugl-Meyer hand gesture recognition for wearable hand rehabilitation robot. https://ieeexplore.ieee.org/document/8489141.

[ref-12] Lyu MX, Lambelet C, Woolley D, Zhang X, Chen WH, Ding XL, Gassert R, Wnderoth N (2020). Comparison of particle filter to established filtering methods in electromyography biofeedback. Biomedical Signal Processing and Control.

[ref-13] Mahdi HJ, Charu P, Muthu BJW (2019). Soft robotic bilateral hand rehabilitation system for fine motor learning. https://doi.org/10.1109/ICORR.2019.8779510I.

[ref-14] Matthew CHC, Jeong HL, Raye CHY (2019). Design and characterization of a soft robotic therapeutic glove for rheumatoid arthritis. Assistive Technology.

[ref-15] Meng W, Liu Q, Zhou ZD, Ai QS (2014). Active interaction control applied to a lower limb rehabilitation robot by using EMG recognition and impedance model. Industrial Robot-An International Journal.

[ref-16] Nycz CJ, Bützer T, Lambercy O, Arata J, Fischer GS, Gasser R (2016). Design and characterization of a lightweight and fully portable remote actuation system for use with a hand exoskeleton. IEEE Robotics and Automation Letters.

[ref-17] Pichiorri F, Morone G, Petti M, Toppi J, Pisotta I, Molinari M, Paolucci S, Inghilleri M, Astolfi L, Cincotti F, Mattia D (2015). Brain-computer interface boosts motor imagery practice during stroke recovery. Annals of Neurology.

[ref-18] Polygerinos P, Wang Z, Galloway KC, Wood RJ, Walsh CJ (2015). Soft robotic glove for combined assistance and at-home rehabilitation. Robotics and Autonomous Systems.

[ref-19] Sangwoo P, Cassie M, Lynne MW, Lauri B, Joel S, Matei C (2018). Multimodal sensing and interaction for a robotic hand orthosis. IEEE Robotics and Automation Letters.

[ref-20] Sheela KG, Deepa SN (2013). Review on methods to fix number of hidden neurons in neural networks. Mathematical Problems in Engineering.

[ref-21] Shi X, Qin PJ, Zhu JQ, Xu SY, Shi WR (2020). Lower limb motion recognition method based on improved wavelet packet transform and unscented kalman neural network. Mathematical Problems in Engineering.

[ref-22] Song R, Tong KY, Hu XL, Li L (2008). Assistive control system using continuous myoelectric signal in robot-aided arm training for patients after stroke. IEEE Transactions on Neural Systems and Rehabilitation Engineering.

[ref-23] Wang JB, Fei YQ, Pang W (2019). Design, modeling, and testing of a soft pneumatic glove with segmented PneuNets bending actuators. IEEE/ASME Transactions on Mechatronics.

[ref-24] Wang DM, Wang YK, Zi B, Cao ZX, Ding HF (2020a). Development of an active and passive finger rehabilitation robot using pneumatic muscle and magnetorheological damper. Mechanism and Machine Theory.

[ref-25] Wang Y, Wu Q, Dey N, Fong S, Ashour A (2020b). Deep back propagation-long short-term memory network based upper-limb sEMG signal classification for automated rehabilitation. Biocybernetics and Biomedical Engineering.

[ref-26] Wu J, Huang J, Wang YJ, Xing KX (2010). A wearable rehabilitation robotic hand driven by PMTS actuators. Intelligent Robotics and Applications.

[ref-27] Yurkewich A, Illya JK, Debbie H, Rosalie HW, Alex M (2020). Hand extension robot orthosis (HERO) grip glove: enabling independence amongst persons with severe hand impairments after stroke. Journal of NeuroEngineering and Rehabilitation.

